# Di-4-pyridyl sulfide–isophthalic acid (1/1)

**DOI:** 10.1107/S1600536808035496

**Published:** 2008-11-08

**Authors:** Jian-Hua Qin, Xiao-Dong Li, Jian-Ge Wang

**Affiliations:** a College of Chemistry and Chemical Engineering, Luoyang Normal University, Luoyang 471022, People’s Republic of China

## Abstract

In the heteromolecular title structure, C_10_H_8_N_2_S·C_8_H_6_O_4_, the two components are linked by O—H⋯N hydrogen bonds to form a one-dimensional chain. These chains are further inter­connected by weak inter­molecular C—H⋯O hydrogen bonds and weak C—H⋯π inter­actions to generate a three-dimensional supra­molecular structure.

## Related literature

For C—H⋯O hydrogen bonds, see: Bhogala *et al.* (2005 ▶); Wang *et al.* (2008 ▶). For C—H⋯π inter­actions, see: Fun & Kia (2008 ▶).
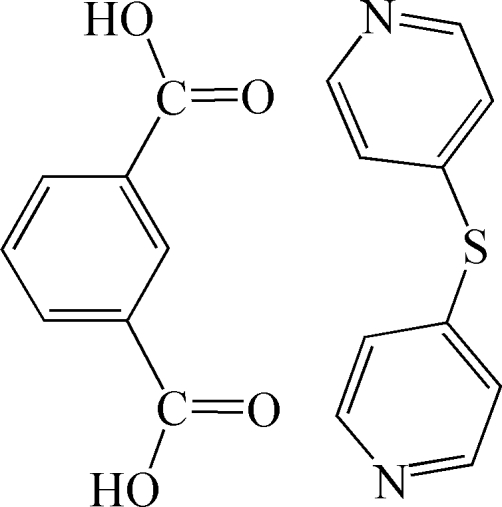


## Experimental

### 

#### Crystal data

C_10_H_8_N_2_S·C_8_H_6_O_4_
*M* *_r_* = 354.37
Triclinic, 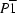

*a* = 6.618 (6) Å
*b* = 8.200 (7) Å
*c* = 16.013 (13) Å
α = 88.808 (11)°
β = 79.340 (11)°
γ = 79.275 (11)°
*V* = 839.0 (12) Å^3^
*Z* = 2
Mo *K*α radiation
μ = 0.22 mm^−1^
*T* = 291 (2) K
0.47 × 0.30 × 0.11 mm

#### Data collection

Bruker SMART CCD area-detector diffractometer
Absorption correction: multi-scan (*SADABS*; Bruker, 1997 ▶) *T*_min_ = 0.905, *T*_max_ = 0.977
6280 measured reflections
3084 independent reflections
1885 reflections with *I* > 2σ(*I*)
*R*_int_ = 0.020

#### Refinement

*R*[*F* ^2^ > 2σ(*F* ^2^)] = 0.080
*wR*(*F* ^2^) = 0.269
*S* = 1.08
3084 reflections
228 parameters
H-atom parameters constrained
Δρ_max_ = 1.05 e Å^−3^
Δρ_min_ = −0.28 e Å^−3^

### 

Data collection: *SMART* (Bruker, 1997 ▶); cell refinement: *SAINT* (Bruker, 1997 ▶); data reduction: *SAINT*; program(s) used to solve structure: *SHELXS97* (Sheldrick, 2008 ▶); program(s) used to refine structure: *SHELXL97* (Sheldrick, 2008 ▶); molecular graphics: *SHELXTL* (Sheldrick, 2008 ▶); software used to prepare material for publication: *SHELXTL*.

## Supplementary Material

Crystal structure: contains datablocks I, global. DOI: 10.1107/S1600536808035496/si2125sup1.cif

Structure factors: contains datablocks I. DOI: 10.1107/S1600536808035496/si2125Isup2.hkl

Additional supplementary materials:  crystallographic information; 3D view; checkCIF report

## Figures and Tables

**Table 1:** Hydrogen-bond geometry (Å, °)

*D*—H⋯*A*	*D*—H	H⋯*A*	*D*⋯*A*	*D*—H⋯*A*
C17—H17⋯O2^i^	0.93	2.45	3.334 (6)	159
C16—H16⋯O2^ii^	0.93	2.58	3.180 (6)	123
C13—H13⋯O4^iii^	0.93	2.31	3.141 (6)	148
C12—H12⋯*Cg*1^iv^	0.93	2.98	3.570 (6)	123
O3—H3*D*⋯N1^v^	0.82	1.83	2.634 (5)	164
O1—H1*D*⋯N2^vi^	0.82	1.84	2.662 (5)	179

Symmetry codes: (i) 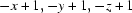; (ii) 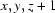; (iii) 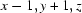; (iv) 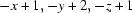; (v) 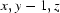; (vi) 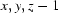. *Cg*1 is the centroid of the C2–C7 isophthalic acid ring.

**Fig. 1.**
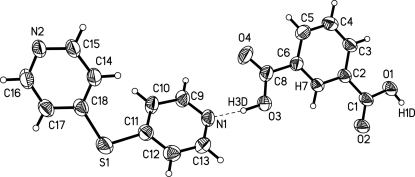
A view of the hetero-molecular components of the title compound with the atom labelling scheme. Displacement ellipsoids are drawn at the 30% probability level.

**Fig. 2.**
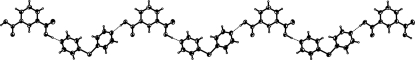
A view of the one-dimensional hydrogen-bond chain motif.

**Fig. 3.**
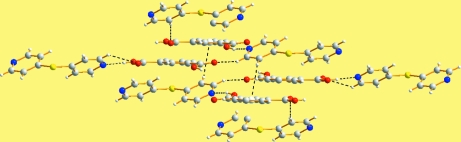
A view of the C—H···O hydrogen bond and the C—H···π interactions in the crystal structure of the title compound.

## supplementary crystallographic information

### Comment

The asymmetric unit consists of one 4,4'-dipyridyl sulfide molecule and one isophthalic acid molecule (Fig. 1). The hetero-molecularar components of (I) are linked by O—H···N hydrogen bonds to form a one-dimensional chain (Table 1 & Fig. 2). These chains interact with each other *via* weak intermolecular C—H···O hydrogen bonds and C—H···π interactions. Within the asymmetric unit, the atoms C13, C16 and C17 act as hydrogen-bond donors (Table 1). The bond lengths and angles of these three hydrogen bonds are comparable with literature data (Bhogala *et al.*, 2005; Wang *et al.*, 2008). These hydrogen bonds, albeit rather weak, link the chains into two-dimensional double layers structure, which are further connected by weak intermolecular C—H···π interactions (Table 1) to generate a three-dimensional supramolecular structure (Fig. 3).

### Experimental

4,4'-dipyridyl sulfide (18.84 mg, 0.1 mmol), isophthalic acid (16.51 mg, 0.1 mmol), and NaOH (8.13 mg, 0.2 mmol) were added to a H_2_O solution (15 ml) in a Teflonlined stainless steel reactor. The mixture was heated at 473 K for 3 d, and then slowly cooled down to room temperature. Colorless crystals of the title compound were obtained.

### Refinement

All hydrogen atoms were positioned geometrically and treated as riding, with C—H bonding lengths constrained to 0.93 (aromatic CH) and O—H bonding lengths constrained to 0.82 (OH), and with *U*_iso_(H) = 1.2*U*_eq_(C) and *U*_iso_(H) = 1.5*U*_eq_(O).

### Crystal data

**Table:**

C_10_H_8_N_2_S·C_8_H_6_O_4_	*Z* = 2
*M**_r_* = 354.37	*F*_000_ = 368
Triclinic, *P*1	*D*_x_ = 1.403 Mg m^−^^3^
*a* = 6.618 (6) Å	Mo *K*α radiation λ = 0.71073 Å
*b* = 8.200 (7) Å	Cell parameters from 1298 reflections
*c* = 16.013 (13) Å	θ = 2.9–21.2º
α = 88.808 (11)º	µ = 0.22 mm^−^^1^
β = 79.340 (11)º	*T* = 291 (2) K
γ = 79.275 (11)º	Block, colorless
*V* = 839.0 (12) Å^3^	0.47 × 0.30 × 0.11 mm

### Data collection

**Table:**

Bruker SMART CCD area-detector diffractometer	3084 independent reflections
Radiation source: fine-focus sealed tube	1885 reflections with *I* > 2σ(*I*)
Monochromator: graphite	*R*_int_ = 0.020
*T* = 291(2) K	θ_max_ = 25.5º
φ and ω scans	θ_min_ = 2.5º
Absorption correction: multi-scan(SADABS; Bruker, 1997)	*h* = −8→7
*T*_min_ = 0.905, *T*_max_ = 0.977	*k* = −9→9
6280 measured reflections	*l* = −19→19

### Refinement

**Table:**

Refinement on *F*^2^	Secondary atom site location: difference Fourier map
Least-squares matrix: full	Hydrogen site location: inferred from neighbouring sites
*R*[*F*^2^ > 2σ(*F*^2^)] = 0.080	H-atom parameters constrained
*wR*(*F*^2^) = 0.269	*w* = 1/[σ^2^(*F*_o_^2^) + (0.1354*P*)^2^ + 0.4871*P*] where *P* = (*F*_o_^2^ + 2*F*_c_^2^)/3
*S* = 1.08	(Δ/σ)_max_ < 0.001
3084 reflections	Δρ_max_ = 1.05 e Å^−^^3^
228 parameters	Δρ_min_ = −0.28 e Å^−^^3^
Primary atom site location: structure-invariant direct methods	Extinction correction: none

### Fractional atomic coordinates and isotropic or equivalent isotropic displacement parameters (Å^2^)

**Table:**

	*x*	*y*	*z*	*U*_iso_*/*U*_eq_	
S1	0.2928 (2)	0.9132 (2)	0.77591 (8)	0.1000 (6)	
O1	0.9948 (5)	0.7101 (5)	0.0723 (2)	0.0868 (10)	
H1D	0.9203	0.7396	0.0371	0.130*	
O2	0.7255 (6)	0.5957 (5)	0.1306 (2)	0.0993 (12)	
O3	0.7222 (6)	0.3409 (5)	0.4098 (2)	0.0958 (12)	
H3D	0.6703	0.3073	0.4560	0.144*	
O4	1.0069 (6)	0.2941 (6)	0.4653 (2)	0.1133 (14)	
N1	0.5677 (7)	1.1790 (5)	0.5430 (2)	0.0733 (10)	
N2	0.7476 (6)	0.8077 (5)	0.9602 (2)	0.0777 (11)	
C1	0.8967 (7)	0.6270 (6)	0.1321 (3)	0.0716 (12)	
C2	1.0110 (6)	0.5765 (5)	0.2024 (3)	0.0636 (10)	
C3	1.2038 (7)	0.6166 (6)	0.2056 (3)	0.0724 (12)	
H3	1.2694	0.6727	0.1605	0.087*	
C4	1.2987 (7)	0.5754 (6)	0.2735 (3)	0.0833 (14)	
H4	1.4264	0.6058	0.2751	0.100*	
C5	1.2062 (7)	0.4887 (6)	0.3400 (3)	0.0772 (12)	
H5	1.2721	0.4597	0.3861	0.093*	
C6	1.0141 (6)	0.4447 (5)	0.3378 (3)	0.0639 (10)	
C7	0.9189 (6)	0.4878 (5)	0.2695 (2)	0.0637 (10)	
H7	0.7912	0.4574	0.2677	0.076*	
C8	0.9164 (7)	0.3505 (6)	0.4099 (3)	0.0734 (12)	
C9	0.6939 (8)	1.0749 (6)	0.5857 (3)	0.0761 (13)	
H9	0.8376	1.0583	0.5661	0.091*	
C10	0.6199 (8)	0.9931 (6)	0.6562 (3)	0.0750 (12)	
H10	0.7120	0.9208	0.6834	0.090*	
C11	0.4099 (7)	1.0177 (6)	0.6868 (3)	0.0691 (11)	
C12	0.2817 (8)	1.1212 (6)	0.6439 (3)	0.0782 (13)	
H12	0.1377	1.1399	0.6631	0.094*	
C13	0.3637 (8)	1.1986 (6)	0.5722 (3)	0.0795 (13)	
H13	0.2728	1.2673	0.5429	0.095*	
C14	0.6067 (8)	0.9811 (6)	0.8561 (3)	0.0833 (14)	
H14	0.6056	1.0787	0.8254	0.100*	
C15	0.7391 (8)	0.9425 (6)	0.9127 (3)	0.0842 (15)	
H15	0.8286	1.0150	0.9182	0.101*	
C16	0.6162 (10)	0.7091 (7)	0.9505 (3)	0.0917 (16)	
H16	0.6169	0.6143	0.9834	0.110*	
C17	0.4795 (8)	0.7377 (6)	0.8952 (3)	0.0855 (15)	
H17	0.3896	0.6644	0.8916	0.103*	
C18	0.4760 (7)	0.8763 (6)	0.8449 (3)	0.0716 (12)	

### Atomic displacement parameters (Å^2^)

**Table:**

	*U*^11^	*U*^22^	*U*^33^	*U*^12^	*U*^13^	*U*^23^
S1	0.0992 (10)	0.1442 (14)	0.0722 (9)	−0.0626 (10)	−0.0181 (7)	0.0271 (8)
O1	0.091 (2)	0.117 (3)	0.0667 (19)	−0.058 (2)	−0.0187 (16)	0.0361 (18)
O2	0.097 (2)	0.150 (3)	0.076 (2)	−0.074 (2)	−0.0345 (18)	0.054 (2)
O3	0.093 (2)	0.138 (3)	0.074 (2)	−0.057 (2)	−0.0301 (18)	0.053 (2)
O4	0.085 (2)	0.152 (4)	0.103 (3)	−0.016 (2)	−0.032 (2)	0.068 (3)
N1	0.095 (3)	0.077 (2)	0.0527 (19)	−0.028 (2)	−0.0184 (19)	0.0174 (17)
N2	0.099 (3)	0.088 (3)	0.056 (2)	−0.045 (2)	−0.0143 (19)	0.0178 (19)
C1	0.074 (3)	0.094 (3)	0.056 (2)	−0.043 (2)	−0.010 (2)	0.018 (2)
C2	0.062 (2)	0.071 (3)	0.059 (2)	−0.021 (2)	−0.0061 (19)	0.0076 (19)
C3	0.069 (3)	0.084 (3)	0.070 (3)	−0.035 (2)	−0.007 (2)	0.011 (2)
C4	0.063 (3)	0.100 (4)	0.092 (3)	−0.031 (3)	−0.013 (2)	0.011 (3)
C5	0.069 (3)	0.089 (3)	0.074 (3)	−0.010 (2)	−0.020 (2)	0.010 (2)
C6	0.061 (2)	0.067 (3)	0.063 (2)	−0.0106 (19)	−0.0114 (19)	0.010 (2)
C7	0.061 (2)	0.073 (3)	0.060 (2)	−0.023 (2)	−0.0099 (19)	0.012 (2)
C8	0.072 (3)	0.082 (3)	0.063 (3)	−0.008 (2)	−0.012 (2)	0.017 (2)
C9	0.076 (3)	0.091 (3)	0.058 (2)	−0.016 (2)	−0.005 (2)	0.016 (2)
C10	0.082 (3)	0.080 (3)	0.057 (2)	−0.007 (2)	−0.008 (2)	0.019 (2)
C11	0.079 (3)	0.076 (3)	0.058 (2)	−0.025 (2)	−0.014 (2)	0.003 (2)
C12	0.071 (3)	0.092 (3)	0.072 (3)	−0.019 (2)	−0.010 (2)	−0.001 (2)
C13	0.084 (3)	0.084 (3)	0.077 (3)	−0.017 (3)	−0.033 (3)	0.012 (3)
C14	0.107 (4)	0.085 (3)	0.072 (3)	−0.043 (3)	−0.027 (3)	0.024 (2)
C15	0.108 (4)	0.096 (3)	0.063 (3)	−0.056 (3)	−0.017 (3)	0.021 (2)
C16	0.136 (5)	0.087 (3)	0.068 (3)	−0.058 (3)	−0.024 (3)	0.022 (2)
C17	0.111 (4)	0.093 (3)	0.068 (3)	−0.059 (3)	−0.019 (3)	0.008 (3)
C18	0.083 (3)	0.086 (3)	0.049 (2)	−0.033 (2)	−0.002 (2)	0.005 (2)

### Geometric parameters (Å, °)

**Table:**

S1—C18	1.767 (5)	C5—H5	0.9300
S1—C11	1.776 (5)	C6—C7	1.369 (6)
O1—C1	1.308 (5)	C6—C8	1.490 (6)
O1—H1D	0.8200	C7—H7	0.9300
O2—C1	1.212 (5)	C9—C10	1.361 (6)
O3—C8	1.302 (6)	C9—H9	0.9300
O3—H3D	0.8200	C10—C11	1.364 (6)
O4—C8	1.198 (5)	C10—H10	0.9300
N1—C13	1.325 (6)	C11—C12	1.356 (6)
N1—C9	1.347 (6)	C12—C13	1.373 (7)
N2—C16	1.323 (6)	C12—H12	0.9300
N2—C15	1.327 (6)	C13—H13	0.9300
C1—C2	1.480 (6)	C14—C18	1.364 (6)
C2—C3	1.385 (6)	C14—C15	1.367 (7)
C2—C7	1.394 (5)	C14—H14	0.9300
C3—C4	1.360 (6)	C15—H15	0.9300
C3—H3	0.9300	C16—C17	1.366 (7)
C4—C5	1.380 (6)	C16—H16	0.9300
C4—H4	0.9300	C17—C18	1.379 (6)
C5—C6	1.391 (6)	C17—H17	0.9300
			
C18—S1—C11	106.3 (2)	N1—C9—H9	118.6
C1—O1—H1D	109.5	C10—C9—H9	118.6
C8—O3—H3D	109.5	C9—C10—C11	119.6 (4)
C13—N1—C9	117.0 (4)	C9—C10—H10	120.2
C16—N2—C15	115.8 (4)	C11—C10—H10	120.2
O2—C1—O1	122.8 (4)	C12—C11—C10	118.0 (4)
O2—C1—C2	122.8 (4)	C12—C11—S1	117.9 (4)
O1—C1—C2	114.3 (4)	C10—C11—S1	124.0 (4)
C3—C2—C7	118.4 (4)	C11—C12—C13	120.2 (4)
C3—C2—C1	122.9 (4)	C11—C12—H12	119.9
C7—C2—C1	118.7 (4)	C13—C12—H12	119.9
C4—C3—C2	121.1 (4)	N1—C13—C12	122.4 (4)
C4—C3—H3	119.5	N1—C13—H13	118.8
C2—C3—H3	119.5	C12—C13—H13	118.8
C3—C4—C5	120.3 (4)	C18—C14—C15	119.8 (4)
C3—C4—H4	119.9	C18—C14—H14	120.1
C5—C4—H4	119.9	C15—C14—H14	120.1
C4—C5—C6	119.8 (4)	N2—C15—C14	123.9 (4)
C4—C5—H5	120.1	N2—C15—H15	118.1
C6—C5—H5	120.1	C14—C15—H15	118.1
C7—C6—C5	119.6 (4)	N2—C16—C17	124.2 (4)
C7—C6—C8	121.2 (4)	N2—C16—H16	117.9
C5—C6—C8	119.2 (4)	C17—C16—H16	117.9
C6—C7—C2	120.9 (4)	C16—C17—C18	119.3 (4)
C6—C7—H7	119.6	C16—C17—H17	120.3
C2—C7—H7	119.6	C18—C17—H17	120.3
O4—C8—O3	122.8 (4)	C14—C18—C17	116.9 (4)
O4—C8—C6	122.5 (4)	C14—C18—S1	124.6 (4)
O3—C8—C6	114.7 (4)	C17—C18—S1	118.4 (4)
N1—C9—C10	122.8 (4)		
			
O2—C1—C2—C3	−178.0 (5)	N1—C9—C10—C11	−1.0 (8)
O1—C1—C2—C3	1.2 (7)	C9—C10—C11—C12	1.5 (7)
O2—C1—C2—C7	0.6 (7)	C9—C10—C11—S1	177.7 (4)
O1—C1—C2—C7	179.8 (4)	C18—S1—C11—C12	−152.0 (4)
C7—C2—C3—C4	−2.1 (7)	C18—S1—C11—C10	31.8 (5)
C1—C2—C3—C4	176.5 (4)	C10—C11—C12—C13	−0.4 (7)
C2—C3—C4—C5	1.6 (8)	S1—C11—C12—C13	−176.8 (4)
C3—C4—C5—C6	−0.6 (8)	C9—N1—C13—C12	1.9 (7)
C4—C5—C6—C7	0.1 (7)	C11—C12—C13—N1	−1.4 (8)
C4—C5—C6—C8	179.8 (4)	C16—N2—C15—C14	−0.5 (8)
C5—C6—C7—C2	−0.6 (7)	C18—C14—C15—N2	−1.3 (8)
C8—C6—C7—C2	179.7 (4)	C15—N2—C16—C17	0.9 (8)
C3—C2—C7—C6	1.6 (6)	N2—C16—C17—C18	0.7 (9)
C1—C2—C7—C6	−177.0 (4)	C15—C14—C18—C17	2.7 (8)
C7—C6—C8—O4	170.8 (5)	C15—C14—C18—S1	178.4 (4)
C5—C6—C8—O4	−8.8 (7)	C16—C17—C18—C14	−2.4 (8)
C7—C6—C8—O3	−12.1 (6)	C16—C17—C18—S1	−178.4 (4)
C5—C6—C8—O3	168.3 (4)	C11—S1—C18—C14	34.9 (5)
C13—N1—C9—C10	−0.7 (7)	C11—S1—C18—C17	−149.5 (4)

### Hydrogen-bond geometry (Å, °)

**Table:**

*D*—H···*A*	*D*—H	H···*A*	*D*···*A*	*D*—H···*A*
C17—H17···O2^i^	0.93	2.45	3.334 (6)	159
C16—H16···O2^ii^	0.93	2.58	3.180 (6)	123
C13—H13···O4^iii^	0.93	2.31	3.141 (6)	148
C12—H12···Cg1^iv^	0.93	2.98	3.570 (6)	123
O3—H3D···N1^v^	0.82	1.83	2.634 (5)	164
O1—H1D···N2^vi^	0.82	1.84	2.662 (5)	179

Symmetry codes: (i) −*x*+1, −*y*+1, −*z*+1; (ii) *x*, *y*, *z*+1; (iii) *x*−1, *y*+1, *z*; (iv) −*x*+1, −*y*+2, −*z*+1; (v) *x*, *y*−1, *z*; (vi) *x*, *y*, *z*−1.
